# Microfeature Segmentation Algorithm for Biological Images Using Improved Density Peak Clustering

**DOI:** 10.1155/2022/8630449

**Published:** 2022-08-18

**Authors:** Man Li, Haiyin Sha, Hongying Liu

**Affiliations:** School of Engineering, Guangzhou College of Technology and Business, Guangzhou 510850, China

## Abstract

To address the problem of low precision in feature segmentation of biological images with large noise, a microfeature segmentation algorithm for biological images using improved density peak clustering was proposed. First, the center pixel and edge information of a biological image were obtained to remove some redundant information. The three-dimensional space of the image is constructed, and the coordinate system is used to describe every superpixel of the biological image. Second, the image symmetry and reversibility are used to obtain the stopping position of pixels, other adjacent points are used to obtain the current color and shape information, and more vectors are used to express the density to complete the image pretreatment. Finally, the improved density peak clustering method is used to cluster the image, and the pixels completed by clustering and the remaining pixels are evenly distributed into the space to segment the image so as to complete the microfeature segmentation of the biological image based on the improved density peak clustering method. The results show that the proposed algorithm improves the segmentation efficiency, segmentation integrity rate, and segmentation accuracy. The time consumed by the proposed biological image microfeature segmentation algorithm is always less than 2 minutes, and the segmentation integrity rate can reach more than 90%. Furthermore, the proposed algorithm can reduce the missing condition and the noise of the segmented image and improve the image feature segmentation effect.

## 1. Introduction

Image segmentation is an important preprocessing technology that has been widely applied to various fields of computer science, especially in the field of biometric images. However, biometric image segmentation is difficult due to the particularity of such images. The quality of image segmentation results determines the quality of image understanding in the next step, which includes the detection and recognition of targets and the relationship between targets in the scene. According to the development history of biometric recognition, the processing of biometric images has always been the focus of research and computer-aided implementation. In this context, many scholars have carried out research on the microfeature segmentation algorithm of biometric images. For instance, Ji et al. [1] proposed the Synthetic Aperture Radar (SAR) image perceptual hash segmentation algorithm, which reduces speckle noise in the first stage and then uses the principal component analysis method to reduce redundant information and obtain smooth segmentation results; Tang and Yu [2] proposed an algorithm for retinal vascular segmentation of color fundus images based on the BP model. The algorithm applies the BP neural network to color fundus image segmentation and uses adaptive histogram equalization, morphological processing, and the matched filtering algorithm to segment the image; Zhuang et al. [3] proposed an ultrasonic image segmentation method based on the fractal theory and fuzzy enhancement. With this method, the ultrasonic image is enhanced by fuzzy technology, and then the image is segmented by enhancement technology; Wang et al. [4] proposed an image segmentation algorithm based on the NSST and vector-valued model. The algorithm combines the NSST and vector-valued model and extracts multidimensional data from the image by using the sampling shear wave transform method to realize image segmentation; Reference [5] proposed a cell image segmentation method based on edge intensity cues. In this method, the edge intensity prompt method is used to recognize the location of leukocytes, and then Grabcut is deployed to segment leukocytes. The above-proposed method can complete the basic segmentation of biometric images. However, it cannot adaptively denoise the noisy biometric images.

To solve this defect, a microfeature segmentation algorithm based on improved density peak clustering is proposed in this paper. The proposed algorithm can reduce the missing condition and the noise of the segmented image and improve the image feature segmentation effect. The contributions of this paper are as follows. (1) The central pixel and edge information of biometric images are obtained, and some redundant information is removed. (2) Image symmetry and reversibility are used to obtain the dwell position of pixels, other adjacent points are used to obtain the current color and shape information, and more vectors are used to express the density with the image preprocessed and the effect of the image feature segmentation optimized. (3) A new clustering algorithm is proposed, which can quickly find the density peak points on the current data and outliers in the dataset and then divide the data points into the nearest classes from the high level to the low level to get the final clustering results.

## 2. Methodology

### 2.1. Image Preprocessing

Generally speaking, if the established space is a three-dimensional (3D) space, three kinds of filtering can be used for processing. The dimension of space is closely related to the type of filtering, that is, the larger the dimension of space [6]. The larger the spatial dimension is, the more filtering is involved, and the difficulty of calculation will be exacerbated, resulting in too many variables and difficulty to control, which will cause image damage and improper preprocessing. Suppose there is a sample point *x*_*i*_, and the edge density before untreated can be expressed as

(1) $$\begin{array}{c} \rho_{i}=\sum d_{ij}-A_{c}, \end{array}$$

where *d*_*ij*_ is the distance between two pixels, *A*_*c*_ refers to the pixel size, and the equation *δ*_*i*_ to calculate the distance between adjacent pixels is

(2) $$\begin{array}{c} \delta_{i}=\left\{\begin{array}{c} \min\left\{d_{ij}\right\}\neq \in I_{S}^{i}\neq_{S}^{i}\neq \emptyset ,\\ \max\left\{d_{ij}\right\}\neq \in I_{S}^{i}\neq_{S}^{i}=\emptyset , \end{array}\right. \end{array}$$

where *I*_*S*_^*i*^ refers to all the point sets, min{*d*_*ij*_} refers to the minimum distance, and max{*d*_*ij*_} refers to the maximum distance.

Based on the above calculation, further processing is carried out. Based on the different pixel points of each part of the image, the density is also different. The distance between two random pixel points is used as the calculation standard, and the gray value of the image is used as the variable; the superpixel density equation of the image is obtained [7].

(3) $$\begin{array}{c} \rho_{i}=\Sigma X\left(d_{ij}-d_{c}\right), \end{array}$$

where *ρ*_*i*_ refers to the density of pixels, *d*_*ij*_ refers to the distance between two random pixels, *d*_*c*_ refers to the distance between pixels after movement, and *X* refers to more than one pixel point. It can be transformed into

(4) $$\begin{array}{c} X\left(x\right)=\left\{\begin{array}{c} 1,\;x\leq 0,\\ 0,\;x>0, \end{array}\right. \end{array}$$

where *x* refers to the quantity. If the edge in the image is too blurred [8] and the color is not easy to distinguish, the equation can be changed to

(5) $$\begin{array}{c} \rho_{i}=\underset{j}{\sum }c^{-1d_{j}/d_{i}}. \end{array}$$

Equations (4) and (5) are based on the clear core information of the image and cannot represent the segmentation processing of all images [3]. If the part of the image meets the segmentation conditions, the image can be processed as follows:

(6) $$\begin{array}{c} \lambda =\rho \cdot \delta , \end{array}$$

where *λ* refers to the coefficient and *δ* refers to the distance between two adjacent pixel points.

If there is too much data, multiple decisions will appear during processing, and multiple decisions will be made at the same time, resulting in confusion in image segmentation [9]. Therefore, it is necessary to relatively reduce some unnecessary variables, build a multidimensional space, describe each superpixel using the coordinate system, and then conduct the calculation. Suppose the space is described as (*l*, *a*, *b*), and any random coordinate is expressed as (*x*, *y*), and then all recognized pixel points correspond to coordinate points one by one. We can get

(7) $$\begin{array}{c} \left\{\begin{array}{c} \left(x,y\right)=\left(x,y\right)\cdot /h_{s},\\ \left(l,a,b\right)=\left(l,a,b\right)./h_{r}, \end{array}\right. \end{array}$$

where *h* refers to the reduction of times, after which the spacing between pixels in the image will become smaller, resulting in an increase in its density. Therefore, if it is detected that the pixel points in the image gradually increase [10], its density can be expressed as

(8) $$\begin{array}{c} \rho_{\text{SP}}=\underset{\text{peSP}}{\sum }p, \end{array}$$

where SP refers to the initial density of superpixels and *p* refers to the quantity.

Then, the stay position of pixel points is obtained by using the symmetry and reversibility of the image [11], and the single feature of each point is found, which is expressed as (*x*, *y*, *l*, *a*, *b*). It can directly describe the main forms of image edge information. Other adjacent points are used to obtain the current color and shape information, and finally, more vectors are used to express the density to complete the image preprocessing.

### 2.2. Image Center Selection Using Improved Density Peak Clustering

**Figure 1:**
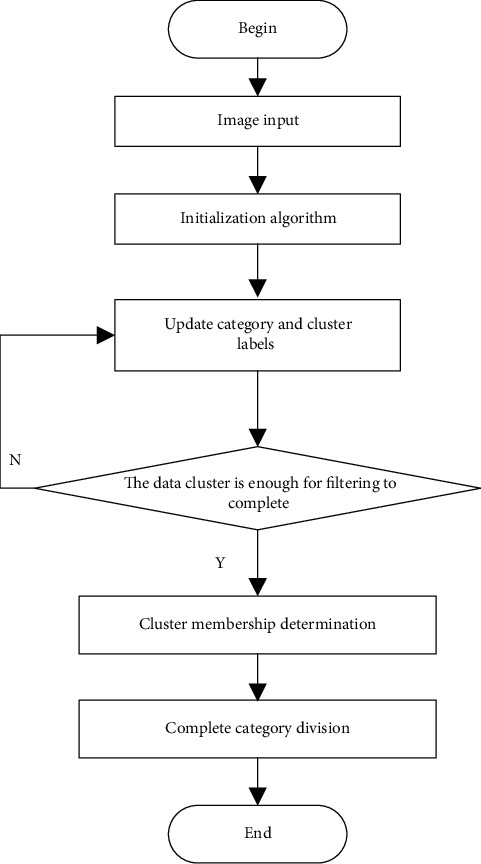
Improved density peak clustering process.

After the above processing, some main information of the image has been mastered. Because the color is easy to distinguish and is not affected by any factors, it can be regarded as a quantitative calculation [12]. There are many remaining density clustering points, so it is necessary to select an optimal clustering center as the variable representative to participate in the density calculation and adopt the improved density peak clustering method in clustering. The process is shown in Figure 1.

The selection of the cluster center should not only consider the quantitative number but also understand the severity of its influencing factors such as noise. The generation of noise will interfere with the image resolution and information transmission. Therefore, the most commonly used denoising method is the two-dimensional entropy denoising method. After the systematic change, the cluster center is finally determined. The expression is shown in

(9) $$\begin{array}{c} \rho_{i}=\underset{j}{\sum }x\left(d_{ij}-d_{c}\right), \end{array}$$

where the function range of *x* is

(10) $$\begin{array}{c} f\left(x\right)=\left\{\begin{array}{c} 1,\;x<0,\\ 0,\;x\geq 0, \end{array}\right. \end{array}$$

where *ρ* refers to the density, *i* and *j* are two adjacent points, and the distance *d*_*c*_ can only be positive and larger than 1. It can be set at will and remain unchanged through the distance equation between two points after clustering [13].

(11) $$\begin{array}{c} \delta_{i}=\left\{\begin{array}{c} \underset{j,p_{i}>\rho_{i}}{\min}\left(d_{ij}\right),\;i\leq 2,\\ \underset{i\geq 2}{\min}\left(d_{ij}\right),\;i=1. \end{array}\right. \end{array}$$

It can be seen from Equation (11) that the density and clustering reach the peak at the same time. At this time, all pixels within the qualified range can be cluster centers, but there cannot be too many cluster centers [12]. Therefore, it is necessary to continue the operation until the cluster center is obtained.

As long as the density of a point is large, it can be included in the screening range. After various considerations and analyses, the density curve can be drawn and further confirmed by the highest point in the graph. Based on the above density and distance, the information entropy equation is obtained after adding the influence of noise [14].

(12) $$\begin{array}{c} \gamma_{i}=\rho_{i}*\delta_{i}, \end{array}$$

where *γ*_*i*_ refers to the information entropy. And the three are in positive proportion, which will increase with the increase of distance and density. Therefore, the entropy values can be sorted in order, the values in the back row can be removed, and the quantity retained is *k*. Then, it is determined by other quantitative comparisons [15]. From the previous data, it can be seen that the gray value of the image will fluctuate regularly within a range without interference from any factors. The range is 0 ~ 255. Therefore, the pixel distance range *d*_*c*_ can be determined as 0 ~ 10, and the change of *k* along with the two is 2 ≤ *k* ≤ 30 . Then, on the basis of information entropy, the variable gray value is added to obtain the mathematical equation:

(13) $$\begin{array}{c} H=\overset{i=0}{\underset{255}{\sum }}p_{i}\log p_{i}, \end{array}$$

where *H* refers to the one-dimensional entropy and *p*_*i*_ refers to the proportion of gray values in the image. The larger the value, the more obvious the color of the representative image, the higher the definition, and the faster the information carried will be extracted [16]. The region of the cluster center is about 2*S* × 2*S*, in which there is more than one pixel point, and different pixel points correspond to different gray values, which poses certain difficulties to segmentation. Therefore, pixels that do not meet the standard can be removed at the same time through multiple calculations [17].

(14) $$\begin{array}{c} D_{s}=d_{lab}+\frac{m}{S}d_{xy}, \end{array}$$

where *d*_*xy*_ refers to the distance between adjacent pixels when gray values are the same, *d*_*lab*_ refers to the distance between adjacent pixels with similar color and different density values, *m* refers to the index, and *S* refers to the point area. The equation is as follows [18]:

(15) $$\begin{array}{c} d_{xy}=\sqrt{{\left(x_{k}-x_{i}\right)}^{2}+{\left(y_{k}-y_{i}\right)}^{2}},\\ d_{lab}=\sqrt{{\left(l_{k}-l_{i}\right)}^{2}+{\left(a_{k}-a_{i}\right)}^{2}+{\left(b_{k}-b_{i}\right)}^{2}}, \end{array}$$

where *l* refers to the color, *a* refers to the image length, and *b* refers to the image width. Therefore, the best distance and density can be determined, and the distance between the cluster center and the edge pixel can be obtained by taking the color as the calculation benchmark:

(16) $$\begin{array}{c} s_{ij}=\left\|c_{i}-c_{j}\right\|, \end{array}$$

where ‖·‖ refers to the actual distance, *c*_*i*_ refers to the spatial characteristic of the pixel point *i*, and *c*_*j*_ refers to the spatial characteristic of the point *j*.

In summary, when the density and distance are maximum, the value of information entropy should be maximized, and the number of superpixels should be small. At this time, several adjacent points are recorded to obtain the cluster center.

### 2.3. Image Feature Segmentation Optimization

After the cluster center is obtained through the constraints of the multidimensional space and vector, the remaining pixels can be evenly distributed into the space [19] so that the distance between each point remains unchanged, and its position remains unchanged, which can be uniformly placed in the grid. On this basis, iteration and addition are carried out to form a fixed circular pattern to quickly segment biometric images.

If the factors interfering with image segmentation remain unchanged and only noise is left, the spatial isolation method can be used to separate the noise from the data information so that the data can be recorded and reduce the impact of noise [20]. Suppose a point in the space is (*x*, *y*), the position of the adjacent pixel point is (*x* + Δ*x*, *y* + Δ*y*), and Δ*x* and Δ*y* are randomly selected in {−1, 0, 1}. However, the prerequisite is (Δ*x*, Δ*y*) ≠ (0, 0). Different spaces will also lead to changes in the distance between pixels. It can be expressed as

(17) $$\begin{array}{c} d_{ij}={\left\|{\left({\bar{x}}_{i}-{\bar{x}}_{j}\right)}^{2}\cdot W\right\|}_{2}, \end{array}$$

where ${\bar{x}}_{i}$ refers to the average threshold value of the adjacent space of *i*, ${\bar{x}}_{j}$ refers to the average threshold value of the adjacent space of *j*, *W* refers to the variance matrix representing threshold in space, and ‖·‖_2_ refers to the times. In order to make the segmentation more thorough without affecting the data collection in other areas, reduce the noise to the greatest extent. However, some details need to be calculated, and small details often affect the global effect. The distance between superpixels is calculated as [21]

(18) $$\begin{array}{c} \delta_{i}=\underset{j:\rho_{j}>\rho_{i}}{\min}\left(d_{ij}\right). \end{array}$$

Redefine the two points as

(19) $$\begin{array}{c} {\text{neigh}}_{i}=j. \end{array}$$

The equation for the pixel with the best performance and the highest density value is

(20) $$\begin{array}{c} \delta_{i}=\underset{j}{\max}\left(d_{y}\right), \end{array}$$

where each pixel exists independently. When the distance is relatively reduced, its density becomes higher, and the maximum density can be obtained in this range [22]. The coordinate points existing in the space are used to accurately segment the region. At this time, the gray value and data information between different regions can be separated:

(21) $$\begin{array}{c} \text{cluste}{\text{r}}_{i}=\exists i:\max\left(\delta_{i}+\rho_{i}\right), \end{array}$$

where (*x*, *y*) are all independent individuals and *n* refers to the coefficient of the 3D space. The algorithm based on the above density peak clustering is improved and optimized, assuming that the preconditions remain unchanged. The mathematical equation is

(22) $$\begin{array}{c} \rho_{i}=\underset{j}{\sum }X\left(d_{ij}-d_{c}\right)P_{i},\\ X\left(d\right)=\left\{\begin{array}{c} 1,\;d<0,\\ 0,\;\text{otherwise}. \end{array}\right. \end{array}$$

It can be seen from Equation ((22)) that the solution of density is actually related to the distance between neighbors, and the image segmentation is actually related to the proportion of gray values. It can not only calculate the density but also see the sparsity of the segmented region. Therefore, it is particularly important to obtain the main features of each pixel. Therefore, a feature function is established [23].

It is assumed that the collected characteristic samples are *X*_1_, *X*_2_, ⋯, *X*_*n*_, and its function is expressed as *f*(*x*), and then the density function is

(23) $$\begin{array}{c} {\hat{f}}_{h}\left(x\right)=\frac{1}{n}\overset{i=1}{\underset{n}{\sum }}K_{h}\left(x-X_{i}\right)=\frac{1}{nh}\overset{i=1}{\underset{n}{\sum }}K\left(\frac{x-X_{i}}{h}\right), \end{array}$$

where *K*(·) refers to the characteristic function, which is usually inversely proportional to the density function, and the constraints are

(24) $$\begin{array}{c} \left\{\begin{array}{c} \int K\left(u\right)du=1,\\ \int uK\left(u\right)du=0,\\ \int u^{2}K\left(u\right)du=u_{2}\left(K\right)>0, \end{array}\right. \end{array}$$

where *u* refers to the constraint coefficient,  *u* = (*x* − *X*_*i*_)/*h* . When all variables in the equation approach 0, the characteristic function is affected first, and the density function will gradually decrease according to its change. At this time, the microfeatures of all points in the image can be obtained clearly, and the damage to the image is minimal.

For the superpixel point *i*, after its space position and occupation ratio are determined and the best distance is obtained, the image microfeature segmentation result can be obtained. The mathematical equation is expressed as

(25) $$\begin{array}{c} \delta_{i}=\left\{\begin{array}{c} \underset{j,\rho_{j}>\rho_{i}}{\min}\left(s_{ij}\right),\;\text{if}\exists j,\;\rho_{j}>\rho_{i},\\ \underset{j}{\max}\left(s_{ij}\right),\;\text{otherwise}. \end{array}\right. \end{array}$$

After several iterations, it can be cut directly to complete the microfeature segmentation of biometric images.

### 2.4. The Proposed Algorithm

**Figure 2:**
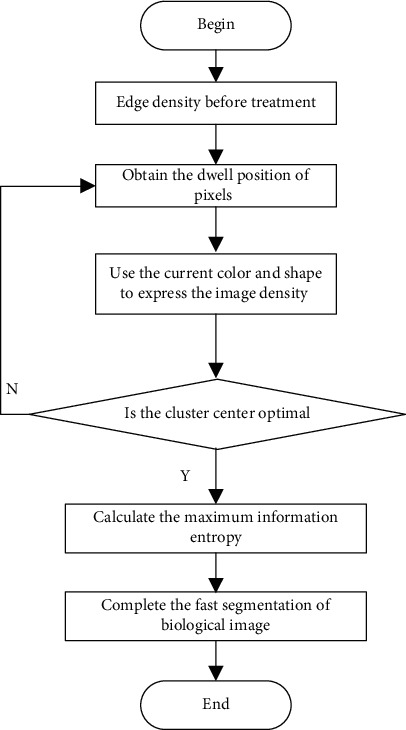
Algorithm implementation process.

The improved density peak clustering method is used to cluster the image, and the clustered pixels and the remaining pixels are evenly distributed into the space to realize the microfeature segmentation of the biological image. The microfeature segmentation algorithm of the biological image based on improved density peak clustering is shown in Figure 2.

## 3. Experimental Analysis and Results

### 3.1. Dataset

To verify the effectiveness of the biometric image microfeature segmentation algorithm based on improved density peak clustering, experiments are carried out. The SSARPHS algorithm proposed by Reference [1], the SBP algorithm proposed by Reference [2], the SSFT algorithm proposed by Reference [3], the SNSSTCV algorithm proposed by Reference [4], and the SEIC algorithm proposed by Reference [5] are compared with the proposed algorithm, and the segmentation effects of the six methods are compared.

The Python development platform is now finished. The simulation data runs on Windows 10, and the algorithm is written in OpenCV. The ImageNet dataset is a vast visual database used in the development of visual object recognition software. ImageNet manually annotates over 14 million picture URLs. ImageNet has over 20,000 categories, such as “balloon” or “strawberry,” each with hundreds of images. The annotation database of the third-party picture URL is available for free from ImageNet. With the Context Dataset Common Objects, all of the image resources in the common objects in the context dataset are linked to the Flickr picture website. The evaluation dataset is partitioned so that 80% of the data is used for training and 20% is used for testing. Initialize the proposed algorithm with the other five segmentation algorithms; store and show the image in BMP format; analyze the image and properly evaluate the experimental findings.

### 3.2. Experimental Index

#### 3.2.1. Image Microfeature Segmentation Time

Compare the image microfeature segmentation time of the biological image microfeature segmentation algorithm, and record the image feature segmentation time of different algorithms.

#### 3.2.2. Gray Image Segmentation Integrity

The visibility of the segmented image is affected by the image's segmentation integrity while segmenting biological images in a microfeature manner. The gray image segmentation integrity rate calculation equation is as follows:

(27) $$\begin{array}{c} R=\frac{\chi^{\prime }}{\chi }\times 100\%, \end{array}$$

where *χ*′ is the number of complete segmentation features of the image and *χ* is the total number of features contained in the image.

#### 3.2.3. Noise after Segmentation

To evaluate the picture's noise, divide it into nonoverlapping image blocks, compute the variance of each image block, rank it from small to large, and select 1% of the total number of blocks to calculate the mean value of the variance, that is, the image's noise variance. The size of image noise is measured in this experiment by the visual noise results in the image.

#### 3.2.4. Missing Condition after Image Segmentation

After applying various algorithms, the lack of image features is the key metric used to assess the algorithm's application performance. The applicability of this method is low after image feature segmentation, resulting in more missing portions.

#### 3.2.5. Segmentation Accuracy

The segmentation accuracy is mainly reflected by the feature segmentation results of the image. The higher the fitting degree between the segmentation results of the image features and the original features, the higher the application accuracy of this method.

### 3.3. Results and Discussion

**Figure 3:**
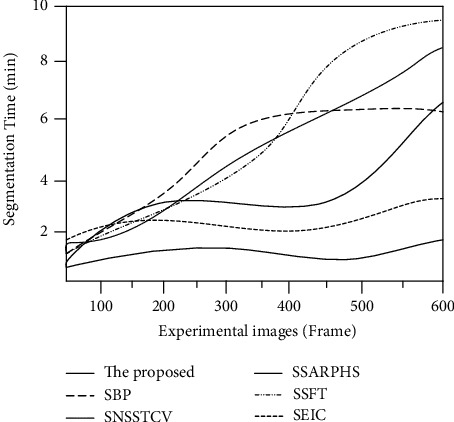
Comparison of segmentation time.

The segmentation time comparison results of the proposed biometric image microfeature segmentation algorithm and the other five segmentation methods are shown in Figure 3.

It is seen from Figure 3 that the feature segmentation time of the SAR image perceptual hash segmentation algorithm proposed by the SSARPHS algorithm is up to 6.5 min, and the faster the segmentation time increases with the increase of the number of experimental images. The segmentation algorithm based on the BP neural network proposed by the SBP algorithm takes up to 8 min, and its time consumed increases linearly. The segmentation algorithm based on the fractal theory proposed by the SSFT algorithm increases rapidly when the number of experimental images is less than 300. When the number of experimental images is more than 300, the feature segmentation time gradually stabilizes and approaches 6 min. Compared with other algorithms, the image segmentation algorithm proposed by the SNSSTCV algorithm takes the most time. When the number of experimental images is 600, the time is 9.5 min. The time consumed by the cell image segmentation algorithm based on edge intensity cues proposed by the SEIC algorithm is closest to that of the proposed method. When the number of experimental images is the highest, the segmentation time is about 2.5 min. The image segmentation algorithm proposed in this study can complete image segmentation in a short time when the number of images is small or large. The other five methods spend less time when there are 10 images. When there are 60 images, the time increases significantly, and the segmentation algorithm based on the BP neural network takes the most time.

**Figure 4:**
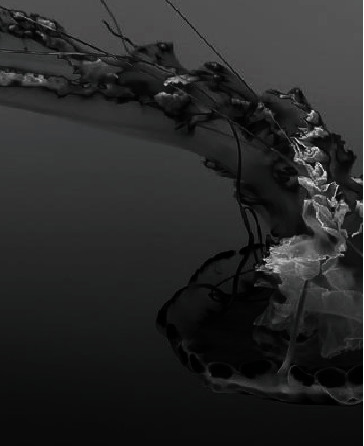
Experimental image.

The segmentation integrity rate of the six methods was compared under the gray image, and the experimental image is shown in Figure 4.

**Figure 5:**
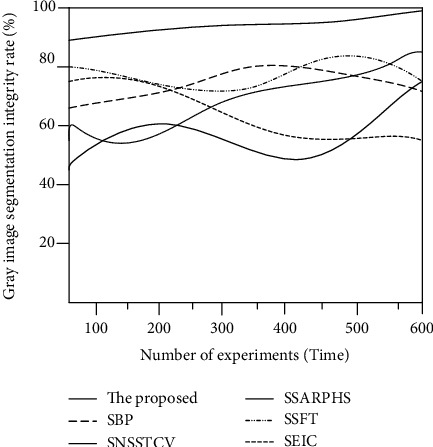
Comparison of gray image segmentation integrity.

The segmentation and comparison results of the six methods are shown in Figure 5.

According to Figure 5, the fluctuation range of the image segmentation integrity rate of the SAR image perceptual hash segmentation algorithm proposed by the SSARPHS algorithm is 40%~70%, and the segmentation integrity test of this algorithm fluctuates greatly with the increase of the number of experimental images. The segmentation integrity rate of the segmentation algorithm based on the BP neural network proposed by the SBP algorithm is up to 75%, and the completion rate of image segmentation decreases with the increase of the number of images. The segmentation algorithm based on the fractal theory proposed by the SSFT algorithm gradually increases the segmentation completion rate to 80% when the number of experimental images is less than 350. When the number of experimental images is more than 350, the image segmentation completion rate gradually decreases to 60%. The image segmentation algorithm based on the NSST and vector-valued C-V model proposed by the SNSSTCV algorithm and the cell image segmentation algorithm based on edge intensity cues proposed by the SEIC algorithm have the greatest fluctuation in segmentation integrity, and the test results are less than 80%. The segmentation algorithm studied has a high segmentation integrity rate, which shows that the proposed method also has a good segmentation effect in the gray image. The other five methods have low segmentation integrity and are greatly affected by the gray image, which is not as good as the algorithm.

**Figure 6:**
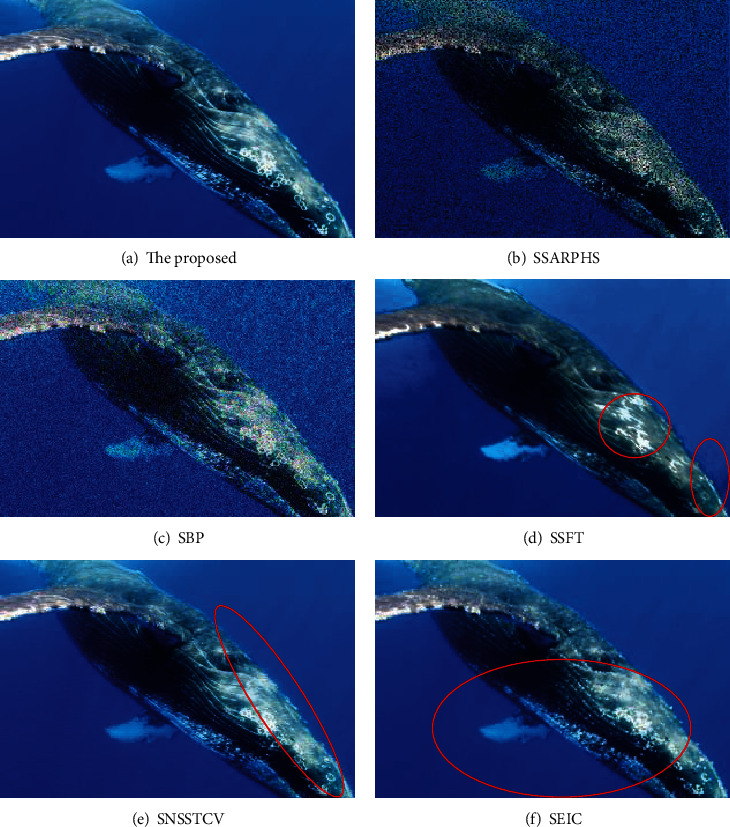
Comparison of image noise after segmentation.

The image noise after segmentation was compared by the six methods, and the results are shown in Figure 6.

It can be seen from Figure 6 that the SAR image perceptual hash segmentation algorithm proposed by the SSARPHS algorithm and the segmentation algorithm based on the BP neural network proposed by the SBP algorithm cannot filter the image noise, and there are obvious noise points, which affect the visual recognition effect of image features. The segmentation algorithm based on the fractal theory proposed by the SSFT algorithm, the image segmentation algorithm based on the NSST and vector-valued C-V model proposed by the SNSSTCV algorithm, and the cell image segmentation algorithm based on edge intensity cues proposed by the SEIC algorithm all have obvious problems of noise points at different positions and cannot clearly show the image. The proposed algorithm has no obvious feature points, and the segmented image quality is high.

**Figure 7:**
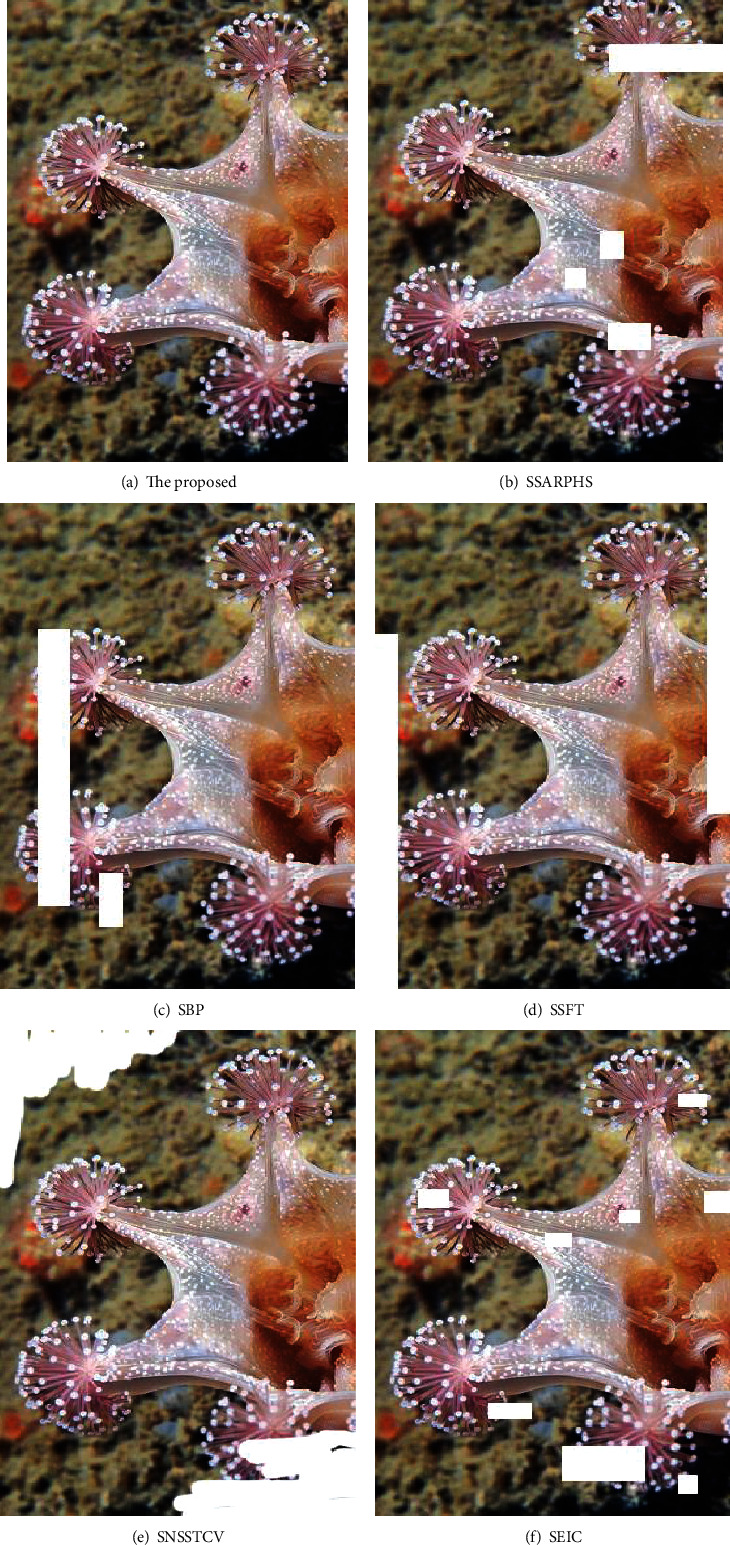
Comparison of missing images after image segmentation.

The missing situation of the images after the segmentation was compared by the six methods, as shown in Figure 7.

As shown in Figure 7, the SAR image perceptual hash segmentation algorithm proposed by the SSARPHS algorithm has many feature deletion problems after feature segmentation. The image feature segmentation results of the segmentation algorithm based on the BP neural network proposed by the SBP algorithm, the image segmentation algorithm based on the NSST and vector-valued C-V model proposed by the SNSSTCV algorithm, and the cell image segmentation algorithm based on edge intensity cues proposed by the SEIC algorithm are not ideal, and the feature deletion is obvious. Although the missing features of the segmentation algorithm based on the fractal theory proposed by the SSFT algorithm appear in the image edge, it also directly affects the image edge recognition and subsequent processing. The biometric image microfeature segmentation algorithm in this study has no image loss after segmentation, and the other five methods have image loss, which may be caused by oversegmentation.

**Figure 8:**
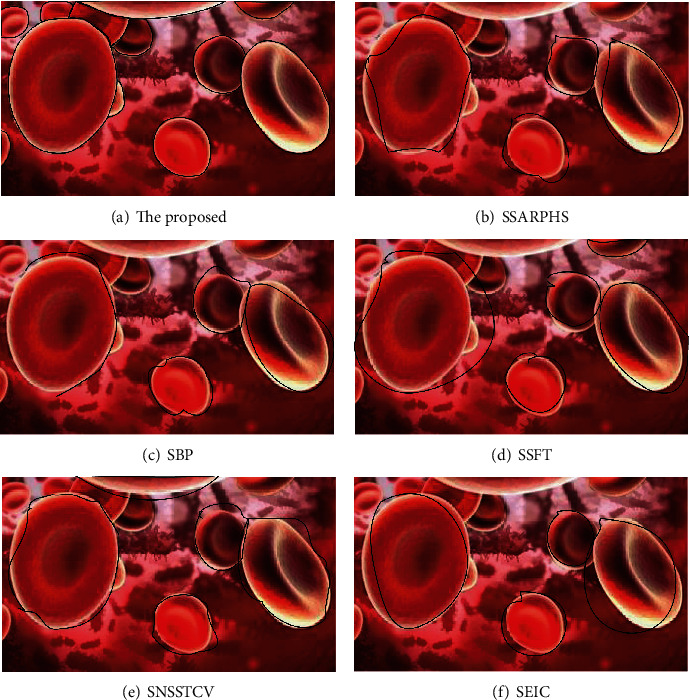
Comparison of segmentation accuracy.

The segmentation algorithm in this paper is compared with the segmentation accuracy of the other five methods, as shown in Figure 8.

Figure 8 shows the comparison of segmentation accuracy. The SAR image perceptual hash segmentation algorithm proposed by the SSARPHS algorithm, the segmentation algorithm based on the BP neural network proposed by the SBP algorithm, the segmentation algorithm based on the fractal theory proposed by the SSFT algorithm, and the image segmentation algorithm based on the NSST and vector-valued C-V model proposed by the SNSSTCV algorithm all have too large, too small, and too few segmentation ranges, and the segmentation accuracy is not as high as the algorithm. In contrast, the algorithm studied can segment all the targets with high accuracy, and there is basically no oversegmentation and less segmentation.

## 4. Conclusions and Future Works

Image segmentation technology is one of the key technologies in image analysis and computer vision, and it is the key to subsequent image processing. Due to the interference of many factors in complex images, there is random noise in addition to the target in the image, and the traditional image segmentation algorithm cannot effectively filter this kind of noise. A microfeature segmentation algorithm based on improved density peak clustering is designed. The algorithm of this research not only improves the segmentation efficiency, segmentation integrity, and segmentation accuracy but also reduces the image noise and image loss after segmentation and effectively solves the current problems. However, there are many factors affecting the characteristics of biometric images, and the research methods do not consider too many factors. In order to further enhance the antinoise performance of the segmentation algorithm, targeted optimization should be carried out from a specific link. Therefore, the influencing factors that may affect image segmentation will be fully considered in future works to improve the effect of image segmentation.

## Figures and Tables

**Algorithm 1:**
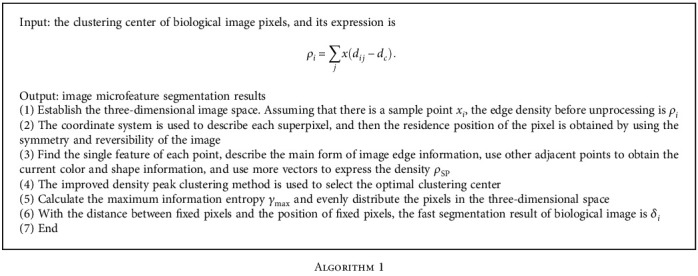


## Data Availability

The data used to support the findings of this study are included within the article.
